# The RNA Polymerase PB2 Subunit of Influenza A/HongKong/156/1997 (H5N1) Restrict the Replication of Reassortant Ribonucleoprotein Complexes

**DOI:** 10.1371/journal.pone.0032634

**Published:** 2012-02-28

**Authors:** Yoko Nakazono, Koyu Hara, Takahito Kashiwagi, Nobuyuki Hamada, Hiroshi Watanabe

**Affiliations:** Division of Infectious Diseases, Department of Infectious Medicine, Kurume University School of Medicine, Fukuoka, Japan; University of Ottawa, Canada

## Abstract

**Background:**

Genetic reassortment plays a critical role in the generation of pandemic strains of influenza virus. The influenza virus RNA polymerase, composed of PB1, PB2 and PA subunits, has been suggested to influence the efficiency of genetic reassortment. However, the role of the RNA polymerase in the genetic reassortment is not well understood.

**Methodology/Principal Findings:**

Here, we reconstituted reassortant ribonucleoprotein (RNP) complexes, and demonstrated that the PB2 subunit of A/HongKong/156/1997 (H5N1) [HK PB2] dramatically reduced the synthesis of mRNA, cRNA and vRNA when introduced into the polymerase of other influenza strains of H1N1 or H3N2. The HK PB2 had no significant effect on the assembly of the polymerase trimeric complex, or on promoter binding activity or replication initiation activity in vitro. However, the HK PB2 was found to remarkably impair the accumulation of RNP. This impaired accumulation and activity of RNP was fully restored when four amino acids at position 108, 508, 524 and 627 of the HK PB2 were mutated.

**Conclusions/Significance:**

Overall, we suggest that the PB2 subunit of influenza polymerase might play an important role for the replication of reassortant ribonucleoprotein complexes.

## Introduction

Influenza A virus is a member of the family *Orthomyxoviridae*, and classified into subtypes by antigenic differences of the two surface glycoproteins, the hemagglutinin (HA) and the neuraminidase (NA) [1]. All 16 HA and 9 NA subtypes are maintained in aquatic birds and can be transmitted to various animals, including humans, pigs, horses and birds. However, avian influenza strains usually do not replicate efficiently in humans, and only three subtypes of H1N1, H2N2 and H3N2 have established sustained infection in human populations since the 1918 Spanish influenza, suggesting a significant restriction of transmission and adaptation of avian strains to humans.

The segmented genome structure of influenza virus facilitates genetic reassortment with other influenza strains which is co-infected in the same cells and has played a pivotal role in the emergence of pandemics. The pandemic strains of the past century have incorporated genes expressing the superficial HA and NA glycoproteins giving rise to new subtypes with novel surface antigens. Mutations of avian HA for acquiring human receptor α2, 6 sialic acid binding specificity is a prerequisite for human adaptation. In addition to acquiring novel genes for superficial glycoproteins, at least one internal gene of the RNA polymerase from avian strains has been concurrently incorporated into human strains. The1957 and 1968 influenza pandemics coincided with the introduction of an avian PB1 gene [2]. Similarly, the 2009 pandemic was caused by a reassortant that acquired PB2 and PA genes from an avian strain [3], [4], [5]. However, the contribution of polymerase genes to pandemic emergence has not been elucidated in detail.

The influenza virus RNA polymerase is a heterotrimeric complex composed of three subunits, PB1, PB2 and PA, which assembles with nucleoprotein (NP) and viral RNA, forming ribonucleoprotein complex (RNP) [6], [7]. The PB1 subunit contains the S-D-D motif characteristic of polymerases and is directly involved in RNA synthesis [8], [9]. The PB2 subunit binds to the cap structure at the 5′ end of host mRNA to generate short capped RNA fragments that are used as primers for viral transcription [6], [10]. The PA subunit plays a role in transcription and replication through its endonuclease activity [11], [12], [13], promoter binding [13], [14], [15], [16], cap binding [13] and possible proteolytic activity [17], [18]. In addition to the central role of the RNA polymerase in RNA synthesis, recent studies have suggested its potential role in genetic reassortment [19], [20], [21], [22]. Artificial hybrid RNA polymerases between human and avian influenza strains often exhibit a functional loss in RNA replication. Reconstitution experiments of influenza RNP in vivo has demonstrated that an introduction of avian PB2 into a background of human strains has often led to a severe reduction of the polymerase activity [23], [24]. It has also been suggested that a specific combination of avian PB2 with human PA or human PB1 leads to a significant decrease in polymerase activity [24], [25]. However, the underlying mechanism that restricts genetic reassortment is still unclear.

In this study, we attempted to characterize and dissect the role of the polymerase genes in giving rise to a functional genetic reassortant. We generated RNP containing hybrid polymerase between human-isolated avian H5N1 and human H1N1 or H3N2. Our results revealed that the PB2 subunit of H5N1 has a strong inhibitory effect on the RNP activity when introduced into the polymerase of other influenza strains. Importantly, H5N1 PB2 could form functional 3P complex properly, but significantly reduced the accumulation of RNP, specifically through the properties of four amino acids in PB2 at position 108, 508, 524 and 627.

## Materials and Methods

### Strains

cDNA clones isolated from the following influenza strains were used: A/HongKong/156/1997 (H5N1) (abbreviated as HK or H), A/Vietnam/1194/2004 (H5N1) (abbreviated as VN or V), A/WSN/1933 (H1N1) (abbreviated as WSN or W), a newly pandemic A/Kurume/K0910/2009 (H1N1) (abbreviated as SW or S), A/NT/60/1968 (H3N2) (abbreviated as NT or N) [26].

### Plasmids

PB1, PB2, PA and NP-expressing plasmids of influenza viruses HK, VN, WSN, SW and NT have previously been described [14], [26], [27]. The plasmid expressing vRNA of NA gene (vNA) from WSN has also been described previously [28]. Mutants of the PB2 plasmids were prepared by site-directed mutagenesis and were fully sequenced.

### RNA isolation and primer extension assay in 293T cell

293T human embryonic kidney cells [12] were transfected with 0.2 µg each of PA, PB1, PB2, NP and vNA expression vector of each strain (WSN, NT, HK, VN or SW) by using Lipofectamine 2000 (Invitrogen). Cells were harvested 30 h posttransfection, and total RNA was isolated with TRIzol reagent (Invitrogen). RNA was then analyzed in a primer extension assay using three primers-one for vRNA, one for mRNA and cRNA, one for 5S rRNA as an internal control [12], [13]. Transcripts were visualized by 7% polyacrylamide gel containing 7 M urea in TBE buffer and were detected by autoradiography. Assays were carried out at least three times with independently transfected cells.

### Preparation of TAP-tagged polymerase and RNP and in vitro assays

For a preparation of the polymerase, 293T cells were transfected with expression vectors containing PB1, PB2 and TAP-tagged PA subunit of each strain. For a preparation of the RNP, NP and vNA expression vectors were also transfected simultaneously. Cells were harvested 2 days posttransfection and the polymerase or RNP was purified by the tandem affinity purification (TAP) method described previously [27]. Partially purified polymerase or RNP was analyzed by 7.5% SDS-PAGE with silver staining (Invitrogen) and confirmed by western blotting with specific antibody against PB1, PB2 and PA [17], . For in vitro assays, the amount of the polymerase was quantitatively adjusted. TAP purification procedure by PA TAP leads to co-purify trimeric complex with PB1-PA TAP heterodimer and PA TAP monomer, and the purified material reveals higher level of PA than other subunits. Adjustments were made by the quantitative measurements of the level of PB2 on silver-stained SDS-PAGE gel, because PB2 indicates the level of trimeric complex. The dinucleotide initiation of replication assay and UV cross-linking to model vRNA and cRNA promoters were performed as reported previously [16], and quantitated by autoradiography and Quantity One software version 4.6.7 (Bio-Rad).

## Results

### Effect of individual polymerase subunits of H5N1 on the RNP activity when reconstituted with other polymerase subunits of H1N1 or H3N2

In order to investigate the role of the RNA polymerase on the restriction of genetic reassortment, we reconstituted reassortant RNP by introducing the polymerase subunit of H5N1 into the polymerase of H1N1 or H3N2. We used two human-isolated H5N1 strains of A/HongKong/156/1997 [HK] and a closely related A/Vietnam/1194/04 [VN], two human H1N1 strains of A/WSN/33 [WSN] and the new pandemic influenza A/Kurume/K0910/09 [SW], and H3N2 strain of A/NT/60/68 [NT], because these strains were extensively analyzed previously [14], [26]. The RNP was reconstituted in human 293T cells from hybrid polymerase on the background of WSN. The steady-state levels of NA reporter mRNA, vRNA and cRNA were measured by primer extension.

**Figure 1:**
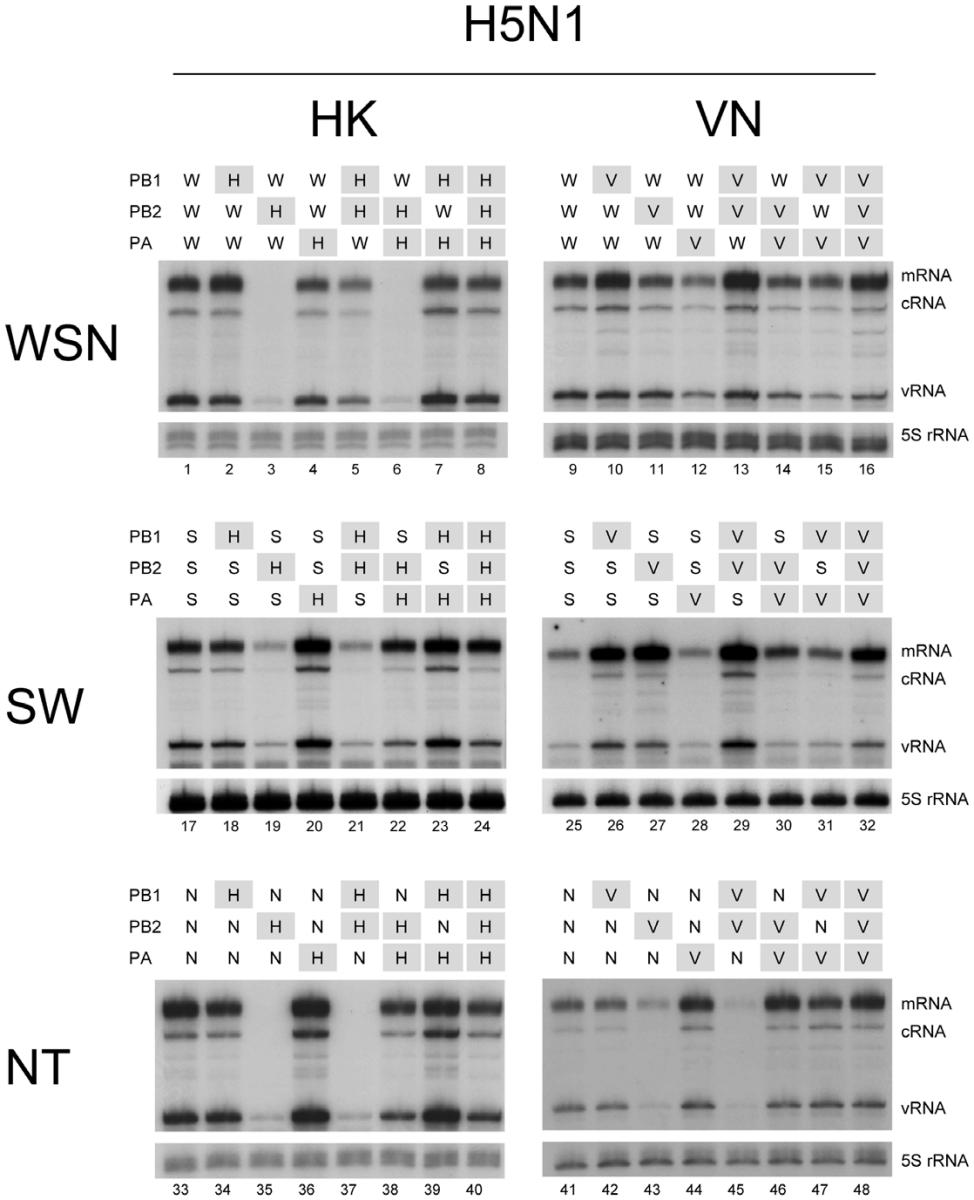
Comparison of RNP activities containing hybrid polymerases between H5N1 strains and human strains in vivo. Polymerase subunits of human strains of A/WSN/1933 (H1N1) (**W**), A/Kurume/K0910/2009 (H1N1) (S) and A/NT/60/1968 (H3N2) (N) were replaced with the corresponding subunits of A/HongKong/156/1997 (H5N1) (H) (left 3 panels) or A/Vietnam/1194/2004 (H5N1) (V) (right 3 panels). The RNP was reconstituted in 293T cells by transfection of plasmids expressing various combinations of polymerase subunits, and WSN derived NP and vNA. Total RNA was extracted 30 h posttransfection. Transcript products of viral mRNA, cRNA and vRNA were measured by primer extension and its positions are indicated on the right. The expected size of mRNA, cRNA, vRNA and 5S rRNA as an internal control are 169–177 nt, 160 nt, 129 nt and 100 nt, respectively.

In the case of the HK strain of H5N1 (Figure 1, left 3 panels), the levels of mRNA, cRNA and vRNA were not significantly affected when HK PB1 alone, HK PA alone, HK PB1 - HK PB2 or HK PB1 - HK PA was introduced into WSN polymerase (Figure 1, lane 2, 4, 5 and 7). However, the RNP activity was severely reduced to essentially inactive levels, with only background levels of input vRNA, when either HK PB2 alone or HK PB2 - HK PA was introduced into WSN polymerase (Figure 1, lane 3 and 6). A similar significant reduction of RNP activities was also observed when either HK PB2 alone or HK PB2 - HK PB1 was introduced into SW (Figure 1, lanes 19 and 21) or NT polymerases (Figure 1, lanes 35 and 37). These results apparently showed that HK PB2 was responsible for the severe reduction of RNP activity, because HK PB2 alone could decrease the activity. This reduction of RNP activity was restored by HK PB1 in WSN polymerase (Figure 1, lane 5), or HK PA in SW or NT polymerase (Figure 1, lanes 22 and 38). On the other hand, in the case of the VN strain of H5N1 (Figure 1, right 3 panels), such remarkable reduction of RNP activity by the PB2 subunit was only observed when introduced into NT polymerase (Figure 1, lanes 43 and 45). VN PB2 did not show the inhibitory effect on either WSN or SW polymerases (Figure 1, lane 11, 14, 27 and 29). On the contrary, VN PB2 alone or VN PB2 - VN PB1 significantly increased the activity of SW polymerase (Figure 1, lanes, 27 and 29). These results indicate that the PB2 subunits of these two H5N1 strains differ in their properties when reconstituted into hybrid RNP with other influenza strains, and HK PB2 has a strong inhibitory effect on RNP activity.

### Rescue of the hybrid RNP activity by mutations in HK PB2

**Figure 2:**
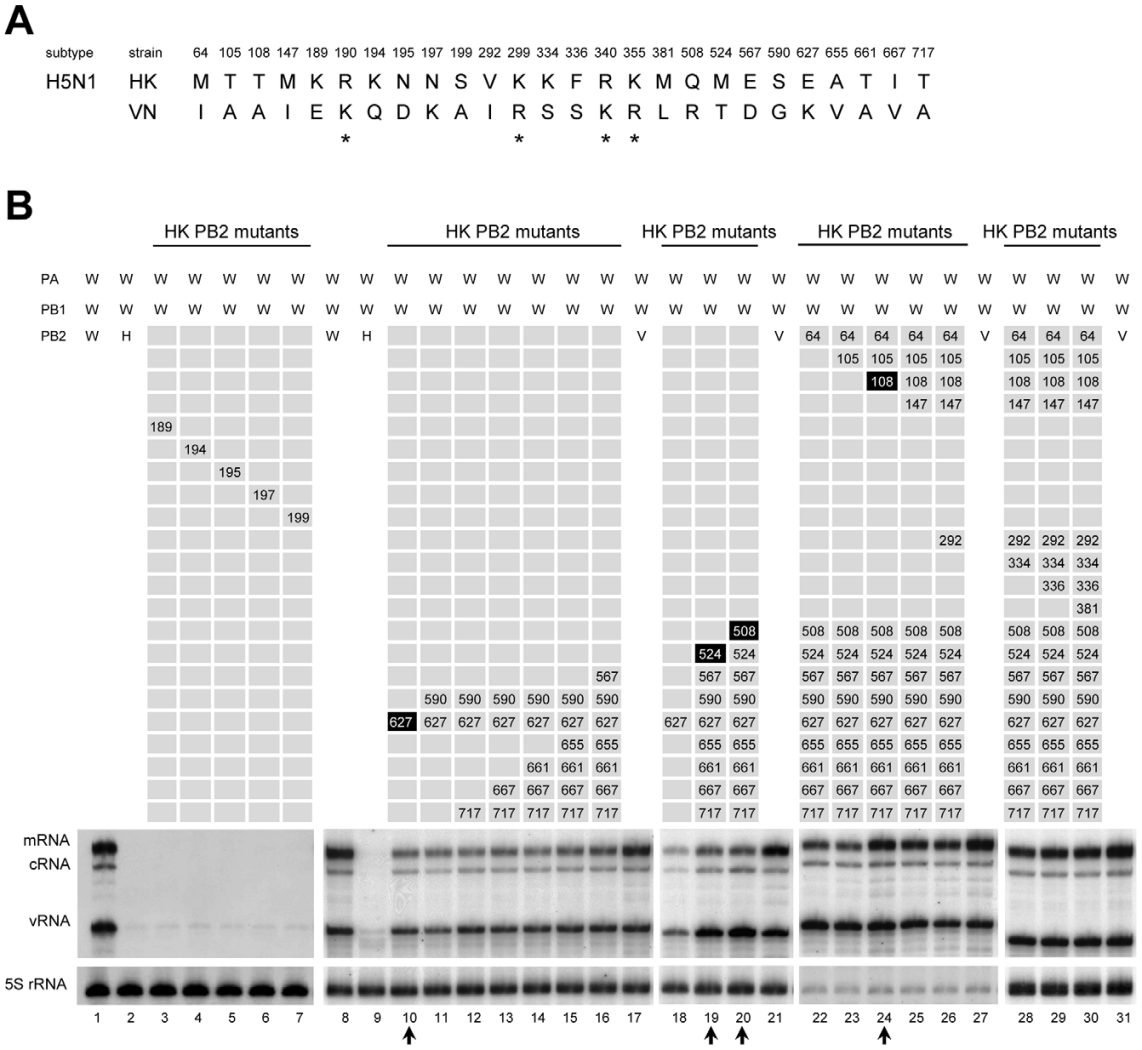
Effects of mutations in the HK PB2 subunit on the impaired RNP activity reconstituted from hybrid polymerase. (**A**) Alignment of amino acid residues of PB2 subunit differing between HK and VN of H5N1 strains. All positions of HK PB2, except for 4 residues (asterisk) that were similar basic amino acids, were sequentially substituted for VN PB2 sequences. (**B**) Steady state level of NA vRNA, mRNA, and cRNA in WSN (W) RNP reconstituted from hybrid polymerase by replacing only PB2 subunit with the HK PB2 wild type (H) (lanes 2 and 9), HK PB2 mutants (lanes 3–7, 10–16, 18–20, 22–26 and 28–30) and VN PB2 (V) (lanes 17, 21, 27 and 31). The numbers in HK PB2 mutants indicate mutated positions in HK PB2. Arrows indicate HK PB2 mutants which showed significant increase of RNP activity. The positions found to be important for the rescue of the RNP activity are highlighted.

Despite originating from the same H5N1 subtype, the amino acid sequence of PB2 between HK and VN differs at 26 positions out of 759 amino acids (Figure 2A). Since HK PB2 showed a severe reduction in RNP activity when introduced into other polymerases as compared to VN PB2, we speculated that a substitution of these 26 amino acids of HK PB2 for the VN PB2 sequence might rescue the impaired RNP activity. To test this, mutations were systemically introduced into HK PB2 at all positions, except for 4 residues at positions 190, 299, 340 and 355 because they were similar basic (R and K) amino acids. Firstly, we focused on residues 189 to 199 because amino acids differences between HK PB2 and VN PB2 were clustered in this region. However, all mutant polymerases K189E, K194Q, N195D, N197K and S199A showed still inactive levels of the RNP activity comparable to WT HK PB2 (Figure 2B, lanes 2 to 7). Next, we focused on residue 627 because PB2 K627 is known to be an important determinant for the virulence and host adaptation of influenza virus. A mutant polymerase E627K of HK PB2 rescued significant activity and the levels of all 3 RNA species (mRNA, cRNA and vRNA) reached about 50% of the VN PB2 activity (Figure 2B, compare lanes 17 with lane 10). In order to attempt to rescue RNP activity further, we then introduced additional mutations into the E627K mutant one by one. Mutations at C-terminal regions did not further increase the RNP activity (Figure 2B, lanes 11–16). However, the additional mutation at M524T significantly increased activity, specifically of cRNA and vRNA, reaching the equivalent level to VN PB2 (Figure 2B, compare lanes 19 with 21). Subsequent mutation at Q508R further slightly increased vRNA to higher levels than VN PB2 (Figure 2B, lane 20), although the mRNA level was still lower than VN PB2. These results suggest a specific role of the positions 508 and 524 on the RNA replication (cRNA and vRNA synthesis), not on transcription (mRNA synthesis). The level of mRNA was fully rescued and the level of all three RNA species nearly reached the VN PB2 level when a mutation was subsequently introduced at T108A (Figure 2B, lane 24). Additional mutations did not increase the RNP activity further (Figure 2B, lanes 25–31). Overall, we conclude that at least 4 positions of 108, 508, 524 and 627 in HK PB2 are responsible for the impaired RNP activity reconstituted from hybrid polymerase.

### Mutations at position 108, 508, 524 and 627 in VN PB2 impair the RNP activity

**Figure 3:**
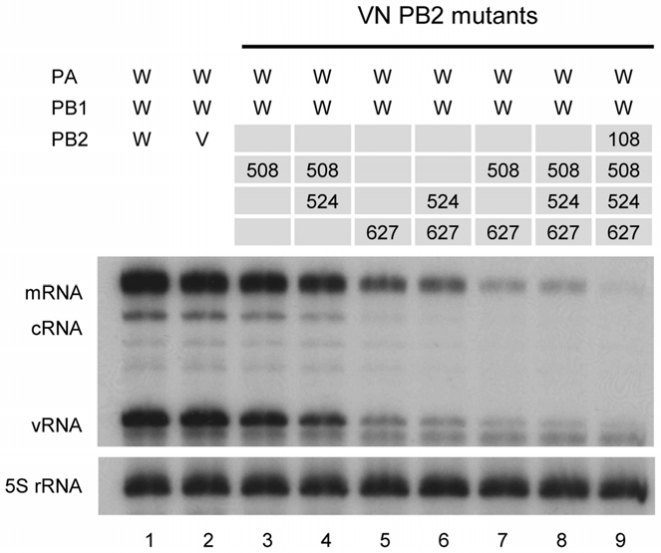
Effects of mutations in the VN PB2 subunit on the RNP activity reconstituted from hybrid polymerase. RNP of WSN (W) was reconstituted from hybrid polymerase by replacing only PB2 subunit with the VN PB2 wild type (V) (lane 2) or VN PB2 mutants (lanes 3–9). The steady state level of vRNA, mRNA, and cRNA was measured by primer extension. The numbers indicate mutated positions in VN PB2.

Since four amino acids in HK PB2 were found to be critical for rescuing activity of the hybrid RNP, it is conceivable that substitution of amino acids at the same position in VN PB2 by the HK PB2 sequence might decrease the RNP activity. Single mutant R508Q or double mutant R508Q/T524M showed a slight decrease in activity compared with VN PB2 (Figure 3, lanes 3 and 4), while single mutant K627E showed a significant decrease in activity to less than 30% of mRNA, and ∼10% of cRNA and vRNA (Figure 3, lane 5). The synthesis of all three RNA species was further decreased in a double mutant R508Q/K627 (Figure 3, lane 7), and markedly reduced to undetectable levels in a fourfold mutant A108T/R508Q/T524M/K627E (Figure 3, lane 9). Taken together, these results support our conclusion that 4 positions at 108, 508, 524 and 627 in HK PB2 are responsible for the optimal activity of RNP reconstituted from hybrid polymerase. We cannot exclude the possibility that residues other than 108, 508, 524 and 627 may also be important for RNP activity, because we have not tested HK PB2 carrying mutations at only these 4 positions.

### HK PB2 impairs neither the assembly of trimeric complex nor the in vitro polymerase activity

**Figure 4:**
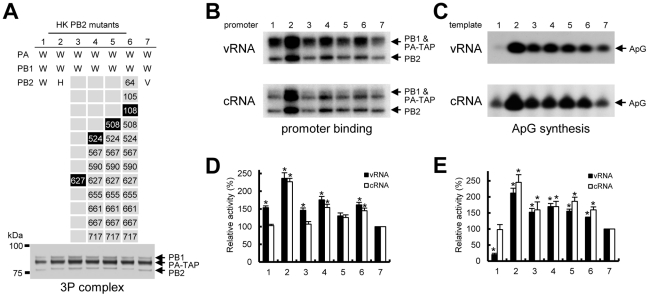
Promoter binding activity and replication initiation activity of HK PB2 mutants. (**A**) Partially purified polymerases analyzed by silver-stained 7.5% SDS-PAGE. Hybrid WSN (W) polymerase replaced only PB2 subunit with HK PB2 wild type (H) (lanes 2), HK PB2 mutants (lanes 3–6) and VN PB2 (V) (lane 7) was transiently expressed in 293T cells and partially purified by using TAP-tagged PA (see materials and methods). The numbers in HK PB2 mutants indicate mutated positions in HK PB2. The highlighted numbers are important for the rescue of the RNP activity. (**B**) UV cross-linking of model vRNA and cRNA promoters to hybrid polymerases. Purified and quantified polymerases were incubated with ^32^P-labelled 3′ strand of the vRNA promoter in the presence of the unlabelled 5′ strand of the vRNA promoter (upper panel), or ^32^P -labelled 3′ strand of the cRNA promoter in the presence of the unlabelled 5′ strand of the cRNA promoter (lower panel). Lane numbers correspond to the numbers in (A). The positions of the cross-linked products are indicated on the right. (**C**) ApG synthesis with a model vRNA promoter (upper panel) or cRNA promoter (lower panel). Lane numbers correspond to the numbers in (A). The position of the specific ApG product is indicated on the right. (**D and E**) Quantification of results obtained in panels B and C, respectively, by phosphorimaging. Data are expressed as percentages relative to VN PB2 (lane 7) (mean ± standard deviation; n = 3). Black bars, model vRNA promoter; white bars, model cRNA promoter. * shows statistical significance at P<0.01 in a Student's t-test.

To address the question why HK PB2 severely inhibited the activity of RNP reconstituted from hybrid polymerase, we initially examined whether HK PB2 affects the correct assembly of the trimeric complex of PB1, PB2, and PA. To allow the purification of trimeric complex, a C-terminally TAP-tagged PA was co-expressed with PB1 and PB2 in 293T cells, and the trimeric complex was affinity purified by a TAP method and quantitatively adjusted (see materials and methods). In the case of HK PB2, the expression level of three subunits was comparable to WSN polymerase or VN PB2 hybrid polymerase (Figure 4A, lane 2), although its RNP activity was impaired. A similar expression level of three subunits was observed in all four HK PB2 mutants having significant RNP activity (Figure 4A, lanes 3–6). Thus, HK PB2 does not appear to affect the correct assembly of the trimeric complex.

An alternative explanation for the defect of RNP activity is a loss of the polymerase activity. The binding of RNA polymerase to the promoter is an essential step to initiate RNA synthesis. Therefore, the promoter binding activity of the polymerase in vitro was assayed by UV cross-linking, then subsequent initiation of RNA synthesis was analyzed by dinucleotide replication assay (see materials and methods). Surprisingly, HK PB2 WT showed a strong activity to bind both vRNA and cRNA promoters (>200% of VN PB2 [Figure 4B and 4D, lane 2]), in spite of the loss of the RNP activity. However, the K627E mutation significantly reduced vRNA and cRNA promoter binding activities to the level of VN PB2 (100–150% of VN PB2 [Figure 4B and 4D, lane 3]). This suggests that position 627 might be involved in promoter binding. Additional mutations did not further affect promoter binding (Figure 4B and 4D, lanes 4–6). The promoter binding activity of the polymerase correlated well with the replication initiation activity and essentially similar results were observed. HK PB2 WT synthesized significant yields of ApG in both vRNA and cRNA templates (>200% of VN PB2 [Figure 4C and 4E, lane 2]). However, K627E mutation remarkably reduced the replication initiation activity (approximately 150% of VN PB2 [Figure 4C and 4E, lane 3]). Additional mutations had little effect on the activity (Figure 4C and 4E, lanes 4–6). Overall these results suggest that the impaired RNP activity by HK PB2 was not due to the defect in the assembly of the polymerase and the loss of the in vitro polymerase activity.

### HK PB2 impairs the accumulation of ribonucleoprotein complex

**Figure 5:**
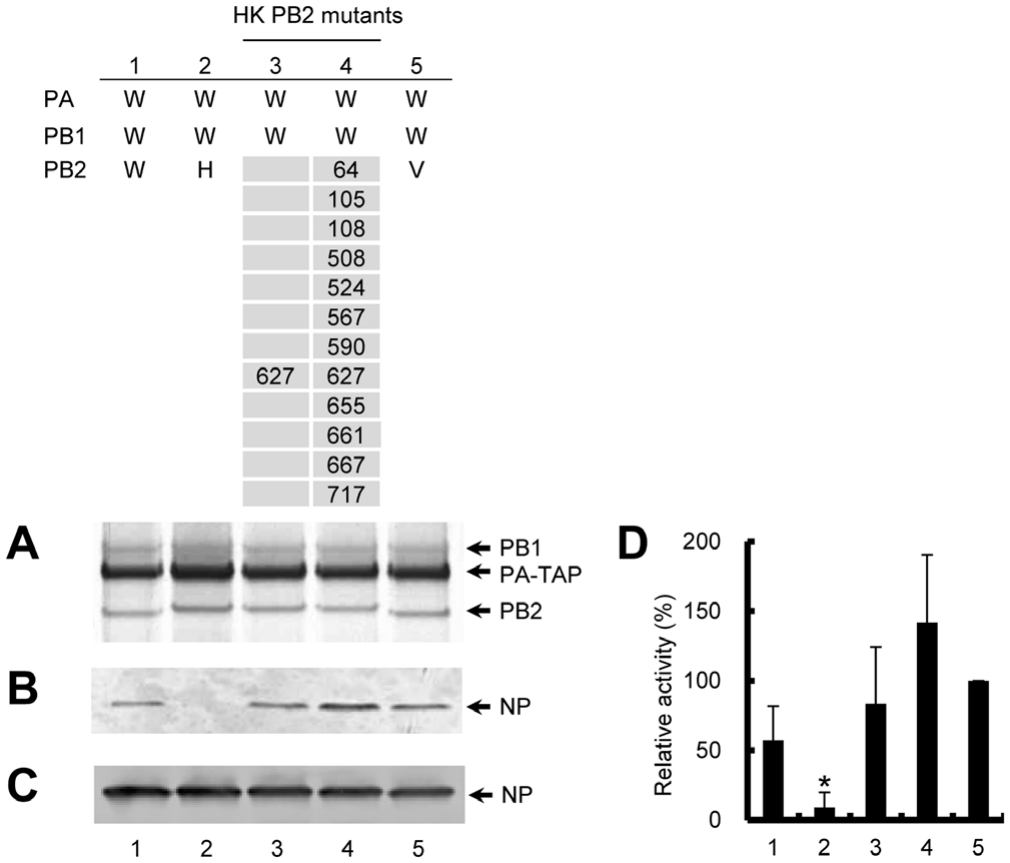
Accumulation of RNP reconstituted from hybrid polymerase. Hybrid RNP was reconstituted in a background of WSN (W) by replacing only PB2 subunit with the HK PB2 wild type (H) (lanes 2), HK PB2 mutants (lanes 3–4) and VN PB2 (V) (lanes 5). Reconstituted RNP in 293T cells was partially purified by using TAP-tagged PA (see materials and methods). The numbers in HK PB2 mutants indicate mutated positions in HK PB2. (**A and B**) Partially purified RNP analyzed by silver-stained 7.5% SDS-PAGE, and by western blotting using specific antibodies for NP, respectively. (**C**) Expression of NP in total cell lysate, analyzed by western blotting using specific antibodies for NP by 7.5% SDS-PAGE. The positions of PB1, PB2, PA-TAP and NP are shown on the right. (**D**) Quantification of results obtained in panel B by phosphorimaging. Data are expressed as percentages relative to VN PB2 (lane 5) (mean ± standard deviation; n = 3). * shows statistical significance at P<0.01 in a Student's t-test.

Another possible explanation for the loss of RNP activity is a defect in the assembly of RNP. To test this possibility, we purified and evaluated the amount of RNP accumulated in vivo. To allow RNP purification, a C-terminally TAP-tagged PA was coexpressed and the reconstituted RNP was affinity purified by a TAP method (see materials and methods). The amount of TAP-purified RNP was measured by Western blotting with specific antibodies for NP (Figure 5B and 5D). Addition of a C-terminal TAP tag at PA did not interfere with the RNP activity (data not shown). In the case of hybrid RNP containing VN PB2, NP was clearly detected, with an essentially similar level to that of WSN PB2 (Figure 5B and 5D, lanes 1 and 5). This indicates that the VN PB2 does not affect in vivo accumulation of RNP. By contrast, in the hybrid containing the HK PB2 (Figure 5A, lane 2) insignificant levels of RNP were detected (Figure 5B and 5D, lane 2), indicating the defect in the replication of RNP. Nevertheless the trimeric complex was correctly formed (Figure 5A, lane 2) and overall NP was expressed at equivalent levels to those seen in the wild-type WSN or VN (Figure 5C, lane 2 compared with lanes 1 and 5). These results clearly showed that HK PB2 had a major inhibitory effect on the accumulation of hybrid RNP. Importantly, the single mutation at 627 rescued significant accumulation of RNP, with an essentially similar level to that of VN PB2 (Figure 5B and 5D, lanes 3 and 5), suggesting that position 627 is obviously involved in the accumulation of RNP. However, the single mutation at 627 did not fully rescue the RNP activity and additional mutation at 108, 508 and 524 were required for the full activity (Figure 2). A significant level of RNP accumulation comparable to that of WSN PB2 or VN PB2 was also observed in the HK PB2 mutant with full RNP activity (Figure 5B and 5D, lane 4). Overall, these results suggested that HK PB2 significantly impairs the accumulation of RNP, explaining the loss of the RNP activity.

## Discussion

The genetic reassortment between two different influenza A viruses can generate 256 (2^8^) genotypes theoretically. However, systematic studies by using reverse genetics have shown that the number of replicative reassortant viruses is apparently limited [23], [25], [30]. Reassortment between A/Thailand/16/04 (H5N1) and A/Wyoming/3/03 (H3N2), or A/equine/Prague/1/56 (H7N7) and A/Yokohama/2017/03 (H3N2), could yield only 63 (254 parent virus) or 29 (256 parent virus) hybrid genotypes, respectively [23], [25]. In this study, we focused on the role of the RNA polymerase on the restriction of genetic reassortment of influenza virus. We constructed artificial hybrid RNPs by replacing the polymerase subunits of H1N1 or H3N2 with the corresponding subunits from two strains of H5N1, HK and VN, and evaluated the RNP activity by measuring steady state levels of mRNA, vRNA and cRNA. We found that the PB2 subunit of HK had a strong inhibitory effect on the activity both in transcription (mRNA synthesis) and replication (cRNA and vRNA synthesis), because it alone could inhibit the activity of all other strains (Figure 1). HK PB1 and HK PA did not appear to have this effect. These results are consistent with the observation that the PB2 subunit of avian or pandemic H1N1 has often lead to a reduction of activity in hybrid polymerase with human strains [23], [24], [31]. Interestingly, the pandemic human H2N2 and H3N2 does not carry avian PB2. Thus, we suggest that an introduction of the PB2 from certain avian H5N1 strains into human strains might be a disadvantage for successful genetic reassortment. Our results cannot exclude the possibility that NP may be involved, because other reassortment studies suggested the importance of NP in host adaptation [30], [32], [33], [34].

Although few studies have described the mechanism of restriction of genetic reassortment, the loss of RNP activity in a hybrid experiment between equine H7N7 and human H3N2 strains was linked to the inability of assembly of the polymerase trimeric complex [25]. On the contrary, the polymerase trimeric complex was properly formed and no significant reduction of the in vitro polymerase activity was seen, even though the RNP activity was impaired when HK PB2 was introduced into the polymerase of other influenza strains (Figure 5). Our results clearly showed that the defect of RNP activity is related to poor accumulation of RNP. The RNP is formed by the association of vRNA to NP and polymerase trimeric complex. We can only speculate that HKPB2 may affect the efficiency of interactions of the polymerase with NP [35], [36], vRNA and cellular factors [37], which are involved in the RNP assembly, thereby affecting RNP accumulation.

**Figure 6:**
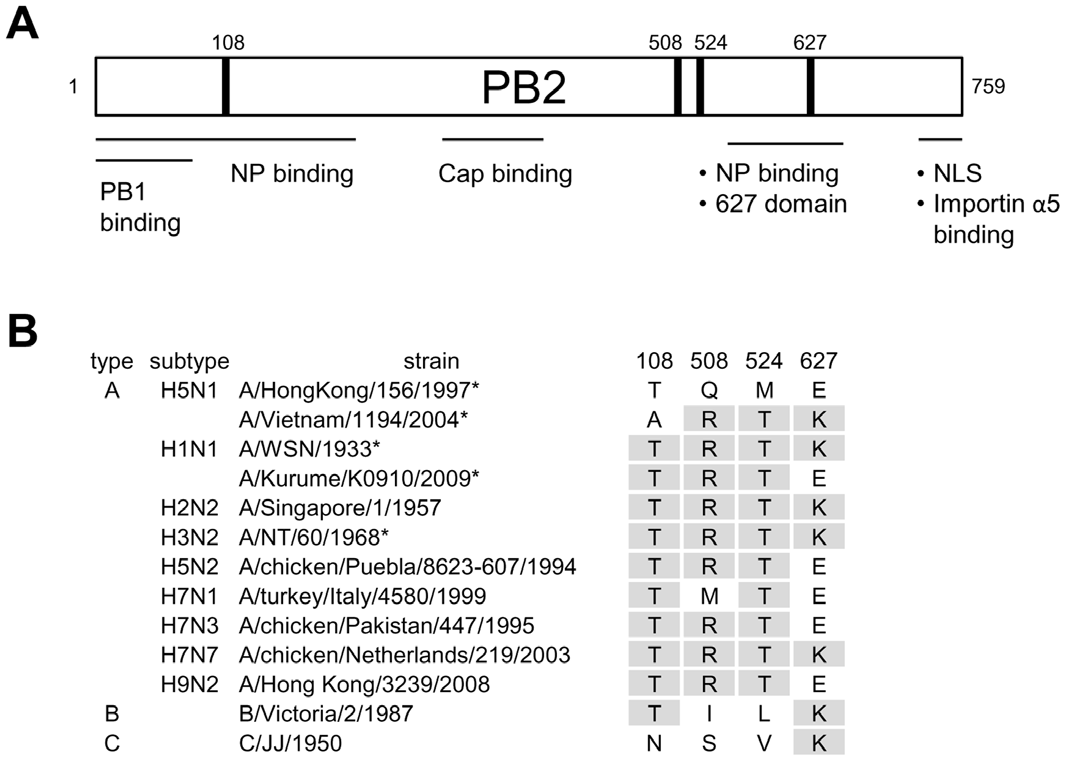
Alignment of PB2 subunit. (**A**) Functional map of PB2 subunit. (B) Alignment of amino acid residues in PB2 which are important for the accumulation of RNP. * shows influenza strains used in this study. Gray shading indicates high evolutionary conservation between influenza A, B and C virus sequences.

The correlation between the presence of lysine at position 627 of PB2 and the efficiency of RNP accumulation has recently been reported for H1N1 strains. The K→E mutation at PB2 627 of human strain A/WSN/1933 (H1N1) significantly decreased the accumulation of RNP reconstituted in human 293T cells and resulted in the concomitant decrease in the RNP activity [39]. In addition, the E→K mutation at PB2 627 of avian strain A/Mallard/Marquenterre/MZ237/1983 (H1N1) in 293T cells increased the accumulation of RNP [19]. These observations are in agreement with our data in H5N1 strains, indicating that the accumulation of RNP was almost fully rescued by the E→K mutation at 627. However, the single mutation at 627 could only partially rescue the RNP activity, and an additional mutation at 108, 508 and 524 was required for full recovery of the RNP activity (Figure 2). Thus, it is possible that amino acid at PB2 627 is mainly involved in the structural assembly of RNP, but other positions 108, 508 and 524 also play an important role in modulating the function of assembled RNP. Position 108 may modulate the interaction with PB1 and preferentially affect transcriptional activity (Figure 2, lane 24), since it is close to the PB1 binding site (Figure 6A) [29], [40]. Positions 508 and 524 are close to cap-binding sites [10], [41] and the 627 domain (538–693) [42]. These positions, we speculate, may affect RNA binding, the function of 627 residue, or may modulate the interaction with host chaperone proteins, such as Hsp90, Hsp70 and CCT [43], [44], [45]. Importantly, an alignment of the PB2 sequences from typical human influenza viruses and avian influenza viruses currently circulating in poultry in some countries, with differing HA and NA subtypes, demonstrated that three positions 108, 508 and 524 were highly conserved among influenza A viruses (Figure 6B). This suggests that these residues are important for the function of RNP in influenza A viruses. In addition, an alignment of HK PB2 sequence with 60 human isolates of H5N1 demonstrated that Q508 and M524 were conserved only less than 2%. Perhaps, these residues might define specific features of HK PB2.

The E→K mutation at HK PB2 627 showed a significant reduction in vRNA promoter and cRNA promoter bindings in cross-linking experiments (Figure 4B). PB2 has been shown to bind both vRNA and cRNA promoters [16], [40], but the precise binding site in PB2 has not yet been identified. Our findings suggest that the PB2 627 is involved in vRNA and cRNA promoter binding. A reduction of the promoter binding is clearly consistent with the reduction of replication initiation activity in vitro (Figure 4C). Structural studies of the C-terminal region of PB2 have shown that the K→E mutation at PB2 627 disrupts the positively charged surface of the protein without altering structure [42]. We propose that the mutation at HK PB2 627 alter the charge of the protein, thereby affecting the promoter binding and subsequent RNA synthesis.

A purified H5N1polymerase shows significantly higher polymerase activity in vitro when compared to human strain A/WSN/33 (H1N1) [14], [46], [47]. The polymerase activity of WSN was increased by the introduction of the PB2 or PA subunit of H5N1. These findings are consistent with our observations that the HK PB2 remarkably enhanced the promoter binding activity and replication initiation activity of WSN polymerase (Figure 4). However, the HK PB2 significantly reduced the polymerase activity in vivo in the RNP reconstitution assay. This different result between in vitro and in vivo is also in agreement with the previous reports [14], [47]. We speculate that a significant reduction of the polymerase activity in vivo, in spite of its strong activity in vitro, could be explained by the poor accumulation of RNP.

Introduction of HK PA into SW and NT polymerases increased the synthesis of mRNA, cRNA and vRNA (Figure 1, lanes 20 and 36). It is also worth noting that HK PA obviously relieved the inhibitory effect of HK PB2 (Figure 1, lanes 22 and 38). This suggests a functional cooperation between PB2 and PA [48]. These results are consistent with recent RNP reconstitution studies suggesting that PA plays a major role in increasing activities of hybrid polymerase [14], [47], [49]. However, such a tendency could not be observed in hybrid WSN polymerase, suggesting that the PA subunit cannot always overcome the restriction of genetic reassortment.

In summary, we have found that the PB2 subunit of influenza A/HongKong/156/1997 (H5N1) has a strong inhibitory effect on the RNP activity when introduced into the polymerase of other influenza strains. In addition, four residues at positions 108, 508, 524 and 627 of the PB2 subunit appear to be important determinants that are involved in the accumulation of functional RNP and in modulating the polymerase activity. These results may suggest a possible mechanism by which the generation of replicative reassortant virus of influenza is highly restricted.
