# Correction: The RNA Polymerase PB2 Subunit of Influenza A/HongKong/156/1997 (H5N1) Restrict the Replication of Reassortant Ribonucleoprotein Complexes

**DOI:** 10.1371/annotation/bcd89872-f3f8-458e-9651-f963a5bedb8a

**Published:** 2012-04-09

**Authors:** Yoko Nakazono, Koyu Hara, Takahito Kashiwagi, Nobuyuki Hamada, Hiroshi Watanabe

There was an error in the title. The correct title is: The RNA Polymerase PB2 Subunit of Influenza A/HongKong/156/1997 (H5N1) Restricts the Replication of Reassortant Ribonucleoprotein Complexes. The correct citation is: Nakazono Y, Hara K, Kashiwagi T, Hamada N, Watanabe H (2012) The RNA Polymerase PB2 Subunit of Influenza A/HongKong/156/1997 (H5N1) Restricts the Replication of Reassortant Ribonucleoprotein Complexes. PLoS ONE 7(2): e32634. doi:10.1371/journal.pone.0032634 

